# Using incidental mark‐encounter data to improve survival estimation

**DOI:** 10.1002/ece3.5900

**Published:** 2019-12-08

**Authors:** Seth M. Harju, Scott M. Cambrin, Roy C. Averill‐Murray, Melia Nafus, Kimberleigh J. Field, Linda J. Allison

**Affiliations:** ^1^ Heron Ecological LLC Kingston ID USA; ^2^ Clark County Desert Conservation Program Las Vegas NV USA; ^3^ U.S. Fish and Wildlife Service Desert Tortoise Recovery Office Reno NV USA; ^4^ San Diego Zoo Global Institute for Conservation Research Escondido CA USA; ^5^ Present address: U.S. Geological Survey Fort Collins Science Center Fort Collins CO USA

**Keywords:** combined datasets, incidental data, mark‐encounter, Mojave desert tortoise, radiotelemetry, survival, translocation

## Abstract

1. Obtaining robust survival estimates is critical, but sample size limitations often result in imprecise estimates or the failure to obtain estimates for population subgroups. Concurrently, data are often recorded on incidental reencounters of marked individuals, but these incidental data are often unused in survival analyses.
2. We evaluated the utility of supplementing a traditional survival dataset with incidental data on marked individuals that were collected ad hoc. We used a continuous time‐to‐event exponential survival model to leverage the matching information contained in both datasets and assessed differences in survival among adult and juvenile and resident and translocated Mojave desert tortoises (*Gopherus agassizii*).
3. Incorporation of the incidental mark‐encounter data improved precision of all annual survival point estimates, with a 3.4%–37.5% reduction in the spread of the 95% Bayesian credible intervals. We were able to estimate annual survival for three subgroup combinations that were previously inestimable. Point estimates between the radiotelemetry and combined datasets were within |0.029| percentage points of each other, suggesting minimal to no bias induced by the incidental data.
4. Annual survival rates were high (>0.89) for resident adult and juvenile tortoises in both study sites and for translocated adults in the southern site. Annual survival rates for translocated juveniles at both sites and translocated adults in the northern site were between 0.73 and 0.76. At both sites, translocated adults and juveniles had significantly lower survival than resident adults. High mortality in the northern site was driven primarily by a single pulse in mortalities.
5. Using exponential survival models to leverage matching information across traditional survival studies and incidental data on marked individuals may serve as a useful tool to improve the precision and estimability of survival rates. This can improve the efficacy of understanding basic population ecology and population monitoring for imperiled species.

## Graphical abstract

Survival estimation using radio‐telemetry datasets is often hampered by small sample size. We found that combining incidental sighting data on marked individuals with radio‐telemetry data improved survival estimation by reducing the spread of 95% credible intervals in all cases with minimal and inconsistent effects on point estimates. In three of eight sub‐groups, survival estimates were only obtainable after inclusion of the incidental encounter data.

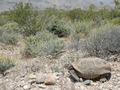

## 1 INTRODUCTION

Robustly estimating survival rates is a fundamental component of understanding basic population ecology and life history theory, and of population conservation and management. Survival rates are a major contributor to population growth or decline and thus, ultimately, population size. Understanding extrinsic factors that influence survival allows population researchers and managers to better understand and manage multiple drivers of survival, such as predation, competition, hunting, and habitat loss. From a conservation perspective, estimating survival rates is a critical part of conserving species populations by understanding which vital rates and extrinsic factors are driving declines or increases in population sizes of imperiled species. However, being imperiled, obtaining sufficient sample sizes for estimating demographic rates such as survival are difficult to obtain, particularly for geographic or demographic subgroups, such as males versus females (e.g., crustaceans, Kordjazi, Frusher, Buxton, Gardner, & Bird, 2016), adults versus juveniles (e.g., snakes, Maida et al., 2018), or individuals from different study sites (e.g., ursids, Howe, Obbard, & Kyle, 2013; e.g., carnivores, Gervasi et al., 2016). In effect, increasing sample sizes can improve estimates of survival (Conner et al. 2015).

A promising solution to the challenge of obtaining sufficient sample sizes for estimating survival rates is the possibility of combining disparate data sources (Dudgeon, Pollock, Braccini, Semmens, & Barnett, 2015; Zipkin & Saunders, 2018). Incidental or opportunistically collected data present a prime opportunity for increasing sample sizes by combining data sources (Jessup, 2003). For many species of conservation concern, widespread attention and effort within a community of biologists across agencies and institutions is spent on documenting incidental sightings of the species, often with individual marking or tagging (e.g., Atlantic shark species, Mejuto, Garcia‐Cortes, & Ramos‐Cartelle, 2005). Many of these incidental sightings of marked individuals are not collected as part of designed studies (hence the term “incidental”) and their occurrences are relegated to spreadsheets and file cabinets. Nonetheless, these data have inherent value because they have minimal or no added cost to collect and contain information on the time between when the animal was first observed and last observed, either alive or dead (Jessup, 2003; Walsh, Dreitz, & Heisey, 2015). Using the appropriate analytical framework, these supplemental data sources have potential to be used alongside “traditional” survival datasets to improve survival estimation.

**Figure 1:**
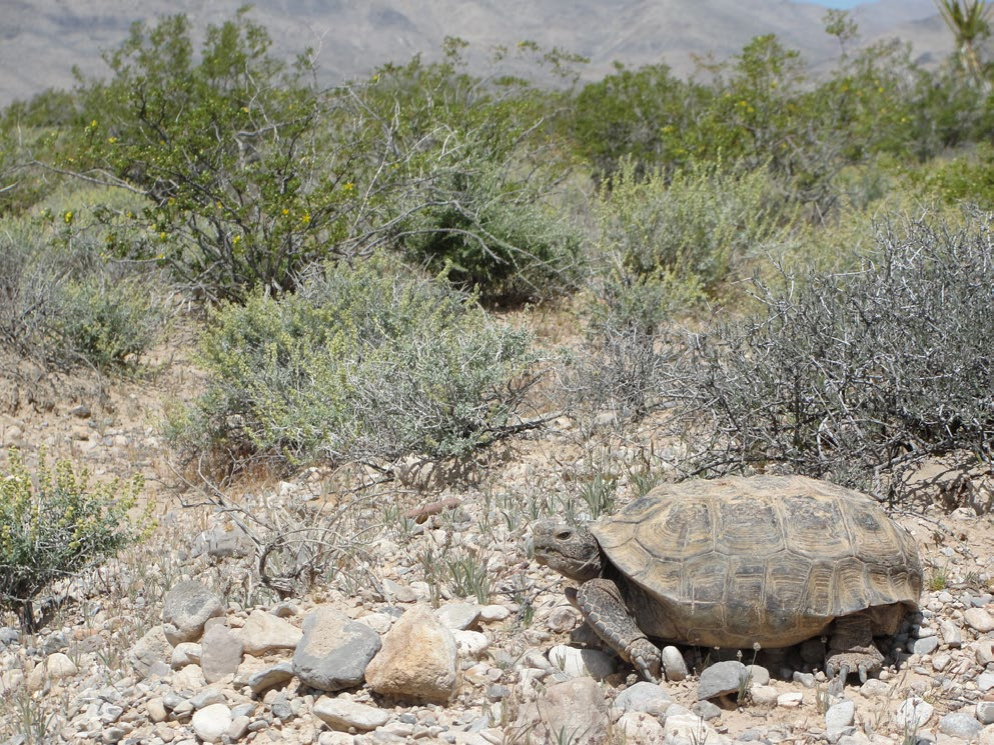
Male desert tortoise (*Gopherus agassizii*) surrounded by Mojave Desert scrub vegetation in southern Clark County, Nevada, USA. Photo credit R. Averill‐Murray

Traditional methods for estimating animal survival are design‐based and come with a variable set of data collection assumptions (Lebreton, Burnham, Clobert, & Anderson, 1992). For example, in a capture‐recapture study, recapture periods, after some defined interval, are used to model and disentangle survival probabilities from recapture probabilities. In contrast, incidental data by definition are not collected under any design assumptions. Information on sampling interval length or sampling area is unavailable because periods when an animal could have been encountered but was not encountered are not recorded. The information that incidental data do have is the span of time between when first encountered and when last encountered. Oftentimes this is the only information present, particularly for rare or elusive species with low encounter rates. Here, we propose the concept of integrating two data sources based on the common information across both datasets, the time from first to last sighting. One of our datasets was collected under a traditional defined sampling scheme (i.e., radiotelemetry), and the other was collected ad hoc with unknown sampling effort (i.e., incidental). The common information across both datasets is continuous time‐to‐event, an established statistical methodology that is increasingly being applied to wildlife survival studies (e.g., Ergon, Borgan, Nater, & Vindenes, 2018). In this paper, we applied a time‐to‐event survival model to combined radiotelemetry and incidental encounter datasets to see if it improved survival estimates for a species of high conservation concern, the Mojave desert tortoise (*Gopherus agassizii*, Figure 1).

Mojave desert tortoise populations are a conservation priority in large parts of the Mojave and Colorado deserts due to widespread population declines, and as a result, they are protected under state and federal regulations (Allison & McCoy, 2014; Allison & McLuckie, 2018; Berry & Aresco, 2014; USFWS, 2011). Tortoises are long‐lived species, meaning that estimation of annual survival rates is important both as a matter of interest and long‐term management (e.g., Curtin, Zug, & Spotila, 2009; Doak, Kareiva, & Klepetka, 1994). Like other tortoise species, Mojave desert tortoises generally have relatively high rates of annual survival, often ranging from 85% to 100% for adults (e.g., Brand et al., 2016; Lovich et al., 2011; Turner, Medica, & Lyons, 1984). Juvenile tortoises, in general, exhibit lower annual survival rates, ranging from 64% to 91% (Bjurlin & Bissonette, 2004; Karl, 1998; Pike, Pizatto, Pike, & Shine, 2008; Zylstra, Steidl, Jones, & Averill‐Murray, 2013). Thus, quantifying survival rates in tortoise populations can be critical to monitoring the magnitude and frequency of high mortality periods, as well as broadly informing conservation and management actions.

We applied our proposed technique to a situation common in ours and other study species: survival following translocation or population augmentation (Mitrus 2005). Initial survival is the first measure to evaluate the success of a translocation (Bell & Herbert, 2017; Miller, Bell, & Germano, 2014), and translocated desert tortoises have had survival rates comparable to those of nearby resident tortoises (Brand et al., 2016; Esque et al., 2010; Field, Tracy, Medica, Marlow, & Corn, 2007; Nussear et al., 2012). For other tortoise species, however, researchers have recorded reduced apparent survival particularly in the first year following translocation (Bertolero, Oro, & Besnard, 2007; Tuberville, Norton, Todd, & Spratt, 2008). Thus, continued quantification of the similarity or difference in survival of resident versus translocated tortoises is of direct relevance to evaluating success and advancing translocation science.

In this study, we evaluated whether survival estimates for Mojave desert tortoises could be improved by supplementing the traditional radiotelemetry‐only dataset with incidentally collected mark‐encounter data. We tested for changes in precision of point estimates and changes in point estimates themselves as potential indicators of bias. We then used the combined dataset to generate survival estimates for demographic and experimental subgroups (e.g., adult vs. juvenile and resident vs. translocated) to evaluate this initial measure of translocation success.

## 2 METHODS

### 2.1 Study area

**Figure 2:**
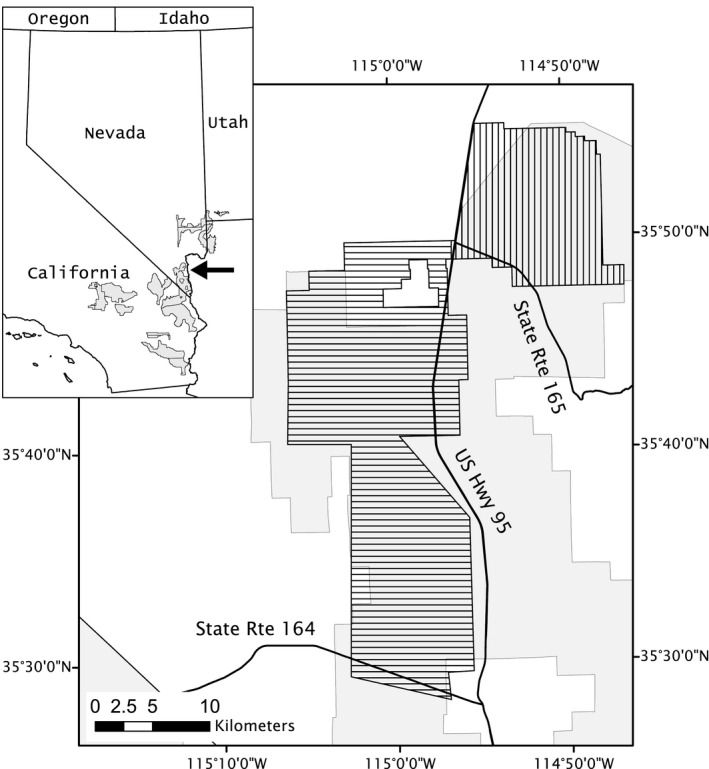
Study area within which radiotelemetry and mark‐encounter data were collected on desert tortoise in the Eldorado Valley (EV), southern Nevada, USA, during 2014–2018. Vertical crosshatching is North EV and horizontal crosshatching is South EV. Gray background in both panels is designated critical habitat for desert tortoise (USFWS, 1994). Arrow in inset locates both EV study areas within larger distribution of critical habitat

Data were collected at two sites within the Eldorado Valley (EV) in southern Nevada, USA (Figure 2). The northern site, North EV, and southern site, South EV, were separated by highways that had been fenced to exclude tortoise egress. Mojave Desert scrub dominated by creosote bush (*Larrea tridentata*) and white bursage (*Ambrosia dumosa*) was the prominent vegetation community at the sites. Generally, the tortoise population in EV had been in decline, though densities varied across the valley (Allison & McLuckie, 2018; USFWS, 2016a).

### 2.2 Translocating and handling desert tortoises

The majority of translocated tortoises were housed at the Desert Tortoise Conservation Center (DTCC) prior to translocation (*n* = 150). The DTCC was a holding facility for tortoises retrieved from private dwellings as pets and from construction sites prior to their development (Field et al., 2007). Tortoises at the DTCC were provided with access to food and water weekly during their active season and were assessed for clinical condition and health on multiple occasions (USFWS, 2016b). Three additional tortoises were translocated directly from construction sites into the Eldorado Valley without being housed at the DTCC. The timeline for releases was 110 tortoises released in September/October 2014, 22 released in April 2015, 3 released in October 2016, and 18 released in September 2017. Those tortoises deemed eligible for release were without clinical signs of communicable disease, in suitable body condition (Lamberski, Braun, & Witte, 2016), and had no other disqualifying conditions according to established protocols (Averill‐Murray, Field, & Allison, 2014; Rideout, 2015; USFWS & Clark County, 2014; USFWS, 2016b). Individuals from the DTCC underwent additional screening that often included longer quarantine periods or more comprehensive health evaluations.

We monitored survival of radio‐telemetered tortoises via weekly relocations from March through October when tortoises are most active above‐ground. Relocations were monthly for adults and twice‐monthly for juveniles from November through February. Upon relocation, the tortoise GPS coordinates and mortality status were determined. If a death was confirmed (e.g., carcass) or inferred (e.g., radiotelemetry unit lying on the ground adjacent to a dug‐up tortoise burrow), the source of mortality was determined where possible. If a radio‐telemetered tortoise was alive when last located, it was treated as right‐censored (e.g., alive for the duration of the study). If a radio‐telemetered tortoise was located dead more than one week after it was last seen alive, the estimated date of death was calculated as the median of the “last alive” and “first dead” observation dates (Mayfield, 1975). By design, the post‐translocation monitoring at the two sites differed (see Averill‐Murray et al., 2014 and USFWS & Clark County, 2014) and the number of tortoises that we relocated was higher in North EV (Table 1).

**Table 1:** Sample sizes for combined Bayesian exponential survival analysis for desert tortoises in Eldorado Valley, Nevada, USA, 2013–2018. All but four mortalities (“Mort.”) were radio‐telemetered tortoises (“Radio”). Each tortoise was monitored for a variable length of time depending on first encounter/ release date, relocation success (for radio‐telemetered tortoises), and reencounter frequency (for mark‐encounter tortoises; “M‐E”)

Study area	Resident						Translocated					
	Adult			Juvenile			Adult			Juvenile		
	Radio^a^	M‐E^b^	Mort.^c^	Radio	M‐E	Mort.	Radio	M‐E	Mort.	Radio	M‐E	Mort.
North	22	10	(2)	0	2	(0)	60	8	(26)	20	1	(5)
South	0	26	(0)	1	7	(1)	15	25	(4)	21	3	(11)

An additional group of 893 un‐telemetered but individually marked wild resident and translocated tortoises were encountered during the study. Of these, 811 were only encountered a single time and were thus excluded from analysis. Therefore, 82 tortoises comprised our mark‐encounter dataset. These tortoises were encountered (resident) or released (translocated) in the study area, individually marked, and then incidentally reencountered at least once in the Eldorado Valley from 2013 to 2018. We refer to these individuals as mark‐encounter tortoises to emphasize the incidental nature of these encounter data so as not to confuse them with traditional mark–recapture and mark–resight study designs (which we did not employ here). Mark–recapture designs require discrete capture occasions, and mark–resight designs involve an initial marking event with resight occasions (White & Burnham, 1999). In contrast, mark‐encounter tortoises were encountered in the study area by field crews conducting work for other desert tortoise projects, including occupancy surveys, line‐distance sampling, and opportunistically during radiotelemetry. Because all reencounters were incidental, there was no discrete marking or reencounter period and the probability of reencounter was unknown across space and time. This means that the right‐censoring itself of the mark‐encounter tortoises was potentially largely due to unknown heterogeneity in field effort which left us unable to spatially delineate sampling sites or temporally delineate sampling occasions. Our approach therefore focused on the unifying piece of information among noncensored and right‐censored radiotelemetry and incidental mark‐encounter tortoises: continuous time passed from first to last sighting (Heisey, Shaffer, & White, 2007).

### 2.3 Evaluating incidental data

Our dataset was a combination of radiotelemetry and mark‐encounter data collected by the Clark County Desert Conservation Program, the U.S. Geological Survey's Henderson Field Office, the San Diego Zoo Global's Institute for Conservation Research, and the U.S. Fish and Wildlife Service's Desert Tortoise Recovery Office. We evaluated the utility of using an exponential survival model to combine the radiotelemetry and mark‐encounter datasets by (a) analyzing only the radiotelemetry dataset and (b) using the same exponential survival model to analyze the combined radiotelemetry and mark‐encounter dataset. We then compared changes in annual survival point estimates and their precision to estimate efficacy and the potential for bias induced by the inclusion of the mark‐encounter data.

We assigned each tortoise to a demographic group depending on whether it was an adult (midline carapace length ≥180 mm) or a juvenile (midline carapace length <180 mm). Males and females were pooled within the adult age group, and sex was undetermined for juveniles. In addition to allowing for group‐specific survival estimates, this grouping allowed us to more finely test the efficacy of incidental mark‐encounter data for survival analysis of population subgroups.

### 2.4 Statistical analysis

To accommodate the radiotelemetry and mark‐encounter data, we used an exponential time‐to‐event statistical model in a Bayesian framework (Colchero & Clark, 2012). We calculated time‐to‐event as the number of weeks between initial capture (for resident tortoises) or release (for translocated tortoises) and the last week observed, with the “event” being the fate of the individual tortoise at last observation. Tortoises that were alive at the last observation were considered right‐censored. We used a simple parametric model to make inference on survival to time *t*, with a random variable for age‐at‐death of *T*, as a function of a hazard rate *λ* (Colchero & Clark, 2012; Lee & Wang, 2003). The hazard rate *λ* was calculated as,

(1) $$\lambda \left(t\right)=\lim_{dt\to 0}\mathrm{Pr}\left(t\leq T<\left(t+dt\right)|t\leq T\right)/dt.$$

Using this constant hazard rate, we generated a survivor function that represented the probability of survival until time *t*,

(2) $$S\left(t\right)=\mathrm{Pr}\left(T\geq t\right)=\exp\left(-\lambda t\right)$$

and calculated the probability density function of age at death,

(3) $$f\left(t\right)=\mathrm{Pr}\left(t\leq T\leq \left(t+dt\right)\right)=S\left(t\right)\lambda t.$$

If all fates were known, data from all individuals would contribute to both likelihood Equations 2 and 3. However, censored individuals only contributed to equation 2 because they provided no information on age‐at‐death to contribute to Equation 3 (Colchero & Clark, 2012). By explicitly identifying individuals that were right‐censored, the full likelihood incorporated the time‐to‐last‐seen data from censored individuals to inform the full exponential likelihood. We note that the exponential time‐to‐event model hazard rate is assumed constant (i.e., not interval specific). Therefore, over the time period of the analysis, all hazard rates, and therefore survival probabilities, are averaged across the time period, weighted by individual observations, even though interval‐specific survival rates would change depending on how the interval is defined (Ergon et al., 2018).

Annual survival estimates were calculated by using a 52‐week period in Equation 2,

(4) $$\text{Ann}\text{.S}\left(t\right)=\exp\left(-52/\left(\lambda_{\left[j\right]}^{-1}\right)\right)$$

for each residency and age class group *j*. Annual survival point and precision estimates were our primary metric for assessing survival and the utility of supplementing the analysis with mark‐encounter data.

We used a log‐linear model to test for residency and age class differences in survival at each study area *k* via the statistical model,

$$\begin{array}{c} \lambda =\exp(\beta_{\text{Res}\text{.Adult}\left[k\right]}+\beta_{\text{Res}\text{.Juv}\left[k\right]}*\text{is}\text{.res}*\text{is}\text{.juv}+\beta_{\text{Tran}\text{.Adult}\left[k\right]}*\text{is}\text{.tran}*\text{is}\text{.adult}\\ +\beta_{\text{Tran}\text{.Juv}\left[k\right]}*\text{is}\text{.tran}*\text{is}\text{.juv}). \end{array}$$

These equations are generally robust to nonobservations of mortalities (i.e., right‐censored individuals that have died) as long as the censoring process occurs at random (Bunck, Chen, & Pollock, 1995). In other words, animals whose fates are not observed during the study period must be censored randomly with respect to mortalities (Ranganathan & Pramesh, 2012). This formulation also explicitly treats each age, translocation status, and study site subgroup as independent from each other. An alternative parameterization could be to introduce a hierarchical prior on some substructure of these subgroups, such as ages across sites or sites across ages. We did not choose this approach because of sample size differences, such that large sample size subgroups would overwhelm estimates from small sample size subgroups. We calculated the censoring rates for the radiotelemetry dataset and the mark‐encounter data to estimate the potential for censoring‐induced bias in the survival estimates (Zhong & Hess, 2009). We also calculated differences in point estimates between radiotelemetry‐only and combined analyses to test for bias.

We used the “is.censored” and “dexp()” functions in package “R2jags” to execute the exponential model while accommodating the right‐censored observations. We used vague normal priors on the coefficients in the exponential model for the λ term. We report survival rates with 95% credible intervals (CrI). We ran the Markov chain Monte Carlo sampler for 1e + 07 draws on 3 independent chains from the posterior. We thinned by every 1e + 03 draw and discarded a burn‐in of 2e + 04 samples to remove high autocorrelation in some of the beta parameters. We achieved effective sample sizes of ~ 1.5e + 04 for each monitored parameter. See Supporting Information for all statistical code and model formulation. All analyses were performed in Program R 3.5.0 (R Core Team, 2018).

## 3 RESULTS

Radiotelemetry and mark‐encounter data spanned the period from March 2013 to April 2018. Most of the tortoises that were part of the full mark‐encounter dataset were excluded from the analysis because they were not reencountered after the initial marking (*n* = 811). The final dataset contained 221 individual tortoises. Of those, 153 were translocatees and 68 were residents (Table 1). There were 139 radio‐telemetered tortoises, 94 of which were alive at the end of monitoring and 45 of which died during the monitoring period. Eighty‐two mark‐encounter tortoises were encountered at least once after initial marking, thus providing information on minimal time survived. Four of the mark‐encounter tortoises were observed as mortalities.

We also calculated the proportion of right‐censored animals to assess the potential for detection probability‐induced bias in survival estimates. The proportion of radio‐telemetered animals that were right‐censored was 0.87 for radio‐telemetered residents and 0.64 for radio‐telemetered translocatees. For mark‐encounter animals, the proportion that was right‐censored was 1.00 for residents and 0.89 for translocatees.

**Figure 3:**
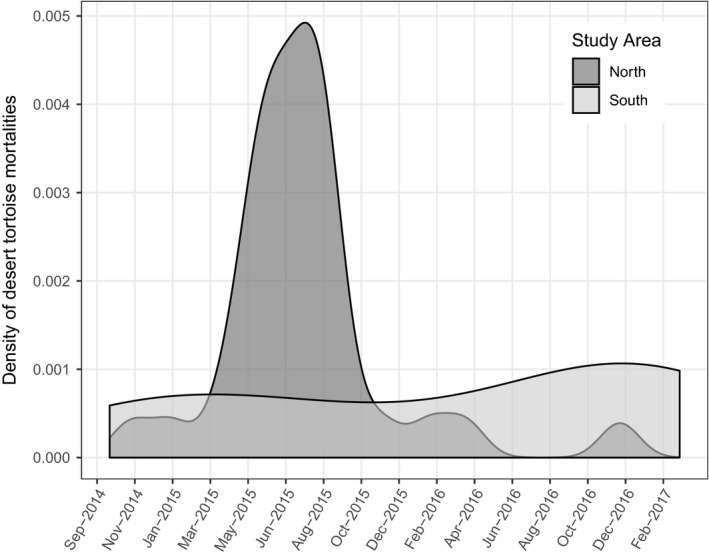
Raw data frequency of desert tortoise mortalities over time in Eldorado Valley, Nevada, USA. Note that the *x*‐axis is truncated to the period of observed mortalities, although the monitoring period extended beyond these dates

The timing of mortalities was not constant across the survey period, with 81.8% (*n* = 27) of all mortalities in the North EV occurring between March and September 2015. No temporal peak in mortality was observed in South EV (Figure 3) and only 12.5% (*n* = 2) of them occurred between March and September 2015. The two resident adult mortalities also occurred during the summer of 2015. At least 21 of the mortalities were the result of predation.

**Figure 4:**
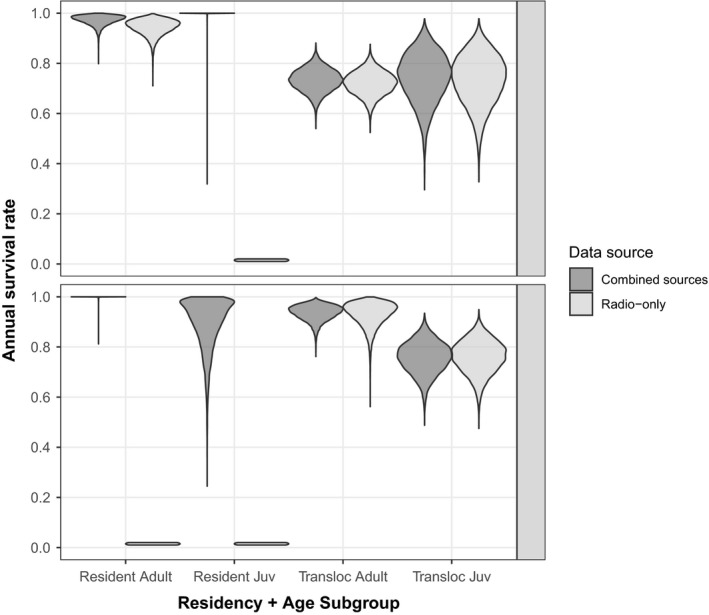
Density of Markov chain Monte Carlo estimated annual survival rates for resident and translocated adult and juvenile desert tortoises in the North and South Eldorado Valley (EV), Nevada, USA, 2013–2017. Data were from the combined radiotelemetry and mark‐encounter dataset (dark gray) and the radiotelemetry‐only dataset (light gray). Radiotelemetry‐only survival rates were inestimable for resident juveniles in both North and South EV and for resident adults in South EV due to small sample sizes

All r‐hat diagnostic values were <1.01, indicating sufficient convergence of the three Markov chain Monte Carlo chains on the same posterior distribution. The combined survival model estimated generally high annual survival rates (e.g., >0.891) for resident adult and juvenile tortoises in both study sites and for translocated adult tortoises in South EV (Figure 4, Table 2). Annual survival for translocated adults in North EV and translocated juveniles in both sites (0.732–0.757) was lower than that of residents. In North EV, translocated adults and translocated juveniles had significantly lower annual survival compared to resident adults (i.e., 95% credible intervals around contrast coefficients did not overlap zero). In South EV, resident juveniles, translocated adults, and translocated juveniles all had significantly lower annual survival compared to resident adults (Figure 4).

**Table 2:** Annual survival rates from the combined data, and increase in precision of annual survival point estimates for desert tortoises in North and South Eldorado Valley, Nevada, USA, 2013–2018. Increase in precision was measured as percent decrease in the spread of 95% credible intervals. Spread of intervals calculated as upper 95% credible interval minus lower 95% credible interval. Percent decrease calculated as [1—combined.spread/radio.spread]. Cells with a “dash” indicate that values were inestimable due to low radiotelemetry sample sizes

Age and translocation by site	Annual survival rates			Spread of 95% CrI		Percent decrease
	Estimate	L 95% CrI	U 95% CrI	Radio‐only	Combined data	
Resident adult—north	0.972	0.922	0.997	0.12	0.075	37.5%
Resident adult—south	0.998	0.981	1.000	–	0.019	–
Resident juv.—north	0.992	0.917	1.00	–	0.083	–
Resident juv.—south	0.891	0.638	0.997	–	0.359	–
Transloc. adult—north	0.732	0.641	0.814	0.184	0.173	6.0%
Transloc. adult—south	0.935	0.861	0.982	0.179	0.121	32.4%
Transloc. juv.—north	0.742	0.537	0.905	0.381	0.368	3.4%
Transloc. juv.—south	0.757	0.626	0.870	0.253	0.244	3.6%

Comparison of survival estimates for the radiotelemetry‐only dataset compared to the combined dataset yielded interesting insights. First, in the radiotelemetry‐only dataset, survival was inestimable for three of the eight possible residency*age class*study site combinations due to small sample size and our independent parameterization of subgroups (*N* = 0 or 1; Table 1). Only via combination of radiotelemetry and mark‐encounter data were we able to generate survival estimates for resident adults in South EV and resident juveniles in both North and South EV. Second, annual survival point estimates were nearly equal between the radiotelemetry‐only and combined datasets, with absolute value differences in point estimates ≤0.029 percentage points (e.g., the greatest difference was 0.943 vs. 0.972). Third, precision of annual survival point estimates was improved in the combined dataset compared with the radio‐only dataset for every residency*age class group we were able to test. The percent decrease in the total spread of the 95% credible intervals ranged from 3.4% to 37.5% (Table 2).

## 4 DISCUSSION

We sought to evaluate the utility of using incidental mark‐encounter data to improve survival estimates under a single continuous time‐to‐event modeling framework. In doing so, we also estimated annual survival rates of desert tortoises in the Eldorado Valley and assessed differences in survival rates for adult, juvenile, resident, and translocated tortoises. We found that in all cases combining incidental mark‐encounter and traditional radiotelemetry data improved the precision of survival estimates for desert tortoises compared to the standalone radiotelemetry dataset. Desert tortoise survival was generally higher for residents versus translocatees, partially due to a large pulse of mortalities for nonresident tortoises during the 5–12 months following a large release of translocated tortoises in one study area. Perhaps most useful was adding the incidental mark‐encounter data to the analysis, allowing an estimate of survival for three out of eight residency, age class, and geographic subgroups that were previously inestimable due to a lack of radiotelemetry samples. Further, addition of the incidental data showed minimal to no evidence for added bias compared to the standard known‐fate dataset.

Wildlife biologists have long seen the value in uniquely marking and recording individual animals whenever possible, as echoed in the call of Jessup (2003) on institutional animal care and use committees to promote collecting incidental and opportunistic data. In survival analyses, such data could help resolve a well‐known sample size problem (Murray, 2006). The precision, and even calculability, of survival estimates is intuitively linked to sample size, as has recently been demonstrated in studies ranging from trout (*Oncorhynchus mykiss*; Conner et al. 2015), rock lobster (*Jasus edwarsii*; Kordjazi et al., 2016), northern Pacific rattlesnake (*Crotalus oreganus*; Maida et al., 2018), to broadnose sevengill sharks (*Notorhynchus cepedianus*; Dudgeon et al., 2015). For species where traditional mark–recapture or radiotelemetry studies are sample size deficient, but where sufficient opportunistic mark‐encounter data exist, time‐to‐event models may be a useful option to bolster survival estimation (Walsh et al., 2015).

The magnitude of the success of the combined data model varied depending on the size of the standalone radiotelemetry dataset. For subgroups with small (*n* ≤ 20) sample sizes of radio‐telemetered tortoises, addition of data from a moderate number of mark‐encounter individuals (*n* ≥ 10) had a substantial impact on reducing the spread of credible intervals with minimal change to point estimates. In contrast, for subgroups with high radiotelemetry sample sizes (e.g., *n* = 60) and/or low sample size of mark‐encounter individuals (e.g., *n* < 5) there was a measurable, albeit minimal, reduction in credible interval spread. This indicates that the approach detailed here may be best suited when radiotelemetry sample sizes are small, and a reasonable amount of supplementary mark‐encounter data are available.

One clear potential risk in the use of either radiotelemetry or incidental mark‐encounter data is the potential for biased survival estimates derived from unequal detection probabilities (DeCesare, Hebblewhite, Lukacs, & Hervieux, 2016; Zhong & Hess, 2009). For radio‐telemetered animals, the risk is minimal if animals can be relocated during each survey effort. For mark‐encounter animals, the potential risk is greater because marked tortoises may be less likely to be encountered once dead due to predation or scavenging. Here, we had high rates of right‐censoring for both detection methods and for residents and translocatees, but with a higher proportion of mark‐encounter individuals (0.95) being right‐censored than radio‐telemetered individuals (0.76). However, as evidence against the presence of bias, we obtained remarkably equivalent point estimates for survival between the radiotelemetry‐only dataset and the combined radiotelemetry and mark‐encounter datasets. The small observed differences in point estimates from both models were both positive and negative, suggesting the lack of systemic bias. Nonetheless, we strongly caution that it remains a potential challenge in other systems and datasets (see DeCesare et al., 2016). Future studies combining known‐fate data with incidental mark‐encounter data would be well served by conducting simulation studies to answer questions on where and how strongly biases may be introduced. We also caution that the potential utility of supplemental data does not replace the need for traditional survival studies and data. Rather, we encourage biologists to incorporate useful supplemental data to improve existing survival studies.

One alternative to our chosen method of using a continuous time‐to‐event model would be imputing missing data for mark‐encounter tortoises between successive periods when the individual was known to be alive (DeCesare et al., 2016). These data could then be analyzed using, for example, Cormack–Jolly–Seber or logistic hazard regression models (DeCesare et al., 2016; Lovich et al., 2014), or perhaps by expanding the width of the resampling “interval” (O'Brien, Robert, & Tiandry, 2005). We viewed this approach as problematic in our case: We did not have information on all sites or dates for all field efforts that were associated with locating incidental mark‐encounter tortoises and thus could not define sample sites or delineate sample periods, and therefore could not generate detection probability models for each of the two datasets (Walsh et al., 2015). Instead, we chose a modeling approach that required fewer assumptions requiring only the length of time between first and last sighting and known fate as input data.

Annual survival was high (>0.97) for resident adults in both study areas and for resident juveniles in North EV but lower (0.732–0.935) for translocated tortoises in both study areas and resident juveniles in South EV. Adult desert tortoises commonly have annual survival rates between 0.90 and 1.00 (Brand et al., 2016; Doak et al., 1994; Esque et al., 2010; Longshore, Jaeger, & Sappington, 2003; Lovich et al., 2011, 2014; Peterson, 1994). Many of the same studies that found generally high survival have noted occasional years where adult tortoise annual survival dropped considerably (e.g., ~18.0%–40.0% mortality in a single year), often in response to regional or prolonged droughts (e.g., Longshore et al., 2003; Lovich et al., 2014).

We found that survival of both adult and juvenile translocated tortoises was lower than that of their resident counterparts. This is contrary to previous Mojave desert tortoise translocation survival studies, which have not found an impact of translocation on short‐term survival (Brand et al., 2016; Esque et al., 2010; Field et al., 2007; Nussear et al., 2012). In other tortoise species, lower apparent survival of translocated tortoises has been reported in up to the first three years post‐translocation (Bertolero et al., 2007; Bertolero, Pretus, & Oro, 2018; Tuberville et al., 2008). We found survival rates for translocated juveniles were equivalent in both study areas and almost 15% lower than survival rates of resident juveniles (albeit resident juveniles had small sample sizes). However, survival of translocated adults in North EV (0.732) was over 20% lower than in South EV (0.935). The higher mortality rate in North EV was primarily driven by the pulse of apparent predation in a single seven‐month span that peaked in the summer of 2015. This suggests that localized spatiotemporal factors, such as weather or predation, were driving the mortality pulse.

Overall, we found an across‐the‐board improvement in estimation of survival of desert tortoise residency, age class, and geographic subgroups by combining radiotelemetry data with incidental mark‐encounter data. Precision of all survival estimates was improved, ranging from a 3.4% to 37.5% reduction in the spread of credible intervals. Survival for three of eight subgroup combinations was inestimable prior to inclusion of the mark‐encounter data. We found minimal to no evidence of bias in survival estimates induced by the mark‐encounter data, although the potential exists for other studies. Survival of translocated desert tortoises was lower than for resident tortoises, primarily due to high mortality during a single seven‐month period in one of our study areas. Together, these findings suggest that using continuous time‐to‐event survival models that incorporate incidental mark‐encounter data could be a useful tool for improving survival estimates of species of conservation focus, including the short‐term success of translocation efforts.

## CONFLICT OF INTEREST

None declared.

## AUTHORS’ CONTRIBUTIONS

SC, RA, MN, KF, and LA conceived the field data collection methodology; SC, MN, KF, and LA collected the data; SH conceived the analysis and analyzed the data; SH led the writing of the manuscript; all authors provided substantial contributions to conceptual and textual development and revisions of the original draft manuscript and have given final approval for publication.

## Data Availability

All input data are archived in the Dryad Digital Repository (https://doi.org/10.5061/dryad.73n5tb2st).
