# Supplementary material for: Using incidental mark‐encounter data to improve survival estimation
Source: Ecol Evol. 2019 Dec 8;10(1):360–70. doi: 10.1002/ece3.5900 (PMC6972812; doi:10.1002/ece3.5900)
Supplement: Supplementary file 1 [file ECE3-10-360-s001.docx]

**Supporting Information for Harju et al. “Using incidental mark-encounter data to improve radio-telemetry survival estimation”**

Sample format for input data.

We used ‘weeks’ as the time interval, although any desired interval can be chosen and should be based on length of monitoring period and general expected survival rates per interval. Note that time-to-event is pooled for both mark-encounter and radiotelemetry data (i.e., both datasets were collapsed to the common piece of information). Right-censored individuals were alive at last sighting, uncensored individuals had observed mortality. See JAGS User Manual for additional instruction on setting up data for use with the is.censored[i] and dinterval() functions.

| ID | weeks  .surv | data  type | wk  .cen | wk  .surv | is  .censored | Study  Area | is  .res | is  .translocated | is  .adult | is  .juv |
| --- | --- | --- | --- | --- | --- | --- | --- | --- | --- | --- |
| 1 | 260 | mark | 260 | NA | 1 | North | 1 | 0 | 1 | 0 |
| 2 | 83 | mark | 83 | NA | 1 | South | 0 | 1 | 1 | 0 |
| 3 | 82 | radio | 82 | NA | 1 | North | 1 | 0 | 1 | 0 |
| 4 | 76 | radio | 76 | NA | 1 | North | 0 | 1 | 1 | 0 |
| 5 | 44 | radio | 262 | 44 | 0 | North | 0 | 1 | 1 | 0 |
| 6 | 46 | radio | 262 | 46 | 0 | North | 0 | 1 | 1 | 0 |
| 7 | 262 | radio | 262 | 262 | 0 | North | 0 | 1 | 1 | 0 |
| 8 | 14 | radio | 262 | 14 | 0 | North | 0 | 1 | 1 | 0 |

*Footnotes:*

weeks.surv = no. of weeks between first and last sighting (= no. days / 7, rounded down)

data type = for tracking purposes, not part of model code

wk.cen = set at weeks.surv for right-censored individuals, for uncensored individuals set at max of weeks.surv of uncensored individuals

wk.surv = set at NA for right-censored individuals, set at weeks.surv for uncensored individuals

is.censored = equals 1 for individuals that were right-censored (i.e., alive at last sighting)

is.res = equals 1 for resident individuals

is.translocated = equals 1 for translocated individuals

is.adult = equals 1 for adult individuals

is.juv = equals 1 for juvenile individuals

Statistical code

Statistical code for Program R for analysis of desert tortoise time-to-event data subject to right-censoring. Data from Code is annotated throughout to facilitate implementation. Comments follow hastag symbols.

# load relevant packages

library(R2jags) # for Bayesian analysis using Program JAGS

library(coda) # for optional processing of JAGS output

# load raw data from a *.csv file; change directory for your file

tortsurv.data <- read.csv("C:/Users/…/Data/exp_survival_tort_rinput.csv")

# write and affix the statistical model for JAGS to reference. See JAGS User

# Manual for instructions on data prep for is.censored[i] and dinterval()

# Model below is formulated for contrast of survival rates of resident

# juveniles, adult translocatees, and juvenile translocatees against resident # adults within each study area. Annual survival estimates are then derived

# for each population subgroup. This specification implicitly assumes

# independent survival rates among population subgroups.

# Note that in JAGS the prior dnorm(0.0,0.001) is parameterized as

# dnorm(mean,precision) where precision = (1/SD^2)

joint.censored.model<- function(){

for(study in 1:n.study){

beta.resadult[study]~dnorm(0.0,0.001)

beta.resjuv[study]~dnorm(0.0,0.001)

beta.tranadult[study]~dnorm(0.0,0.001)

beta.tranjuv[study]~dnorm(0.0,0.001)

lambda.resadult[study]<- exp(beta.resadult[study])

lambda.resjuv[study]<- exp(beta.resadult[study]+beta.resjuv[study])

lambda.tranadult[study]<- exp(beta.resadult[study]+beta.tranadult[study])

lambda.tranjuv[study]<- exp(beta.resadult[study]+beta.tranjuv[study])

mu.resadult[study]<- 1/lambda.resadult[study]

mu.resjuv[study]<- 1/lambda.resjuv[study]

mu.tranadult[study]<- 1/lambda.tranadult[study]

mu.tranjuv[study]<- 1/lambda.tranjuv[study]

annsurv.resadult[study]<- exp(-52/mu.resadult[study])

annsurv.resjuv[study]<- exp(-52/mu.resjuv[study])

annsurv.tranadult[study]<- exp(-52/mu.tranadult[study])

annsurv.tranjuv[study]<- exp(-52/mu.tranjuv[study])

}

for(i in 1:n.all){

is.censored[i] ~dinterval(wk.surv[i],wk.cen[i])

wk.surv2[i]~dexp(lambda[i])

lambda[i] <- exp(beta.resadult[study[i]] + beta.tranadult[study[i]]*is.translocated[i]*is.adult[i] + beta.resjuv[study[i]]*is.res[i]*is.juv[i] + beta.tranjuv[study[i]]*is.translocated[i]*is.juv[i])

}

}

# set the stochastic parameters from the model that JAGS should monitor

jags.joint.parms <- c("mu","beta.resadult","beta.resjuv","beta.tranadult","beta.tranjuv","annsurv.resadult","annsurv.resjuv","annsurv.tranadult","annsurv.tranjuv")

# create a list of data pieces referenced in the model statement. JAGS can’t

# see the whole R workspace, everything used needs to be put in this list

jags.joint.data <- list(wk.cen=tortsurv.data$wk.cen, wk.surv=tortsurv.data$wk.surv, is.censored = tortsurv.data$is.censored, n.all = 221, is.translocated=tortsurv.data$is.translocated, is.juv=tortsurv.data$is.juv, is.res=tortsurv.data$is.res, is.adult=tortsurv.data$is.adult, study=tortsurv.data$StudyArea, n.study=2)

# run the model statement, referencing data, parms, inits, and

# mcmc sampling specifications

# this model updates efficiently without inits, but they may be

# required in other datasets or model specifications

dt.joint.survival.fm <- jags(data=jags.joint.data, parameters.to.save=jags.joint.parms, n.iter=10000000,n.burnin=20000,n.thin=2000, inits=NULL,model.file=joint.censored.model)

# call summary results from model run

dt.joint.survival.fm
